# Ethyl α-l-sorboside

**DOI:** 10.1107/S2414314620016259

**Published:** 2020-12-18

**Authors:** Natsumi Nagayama, Norito Taniguchi, Mao Matsumoto, Kei Takeshita, Tomohiko Ishii

**Affiliations:** a Department of Advanced Materials Science, Faculty of Engineering, Kagawa University, 2217-20 Hayashi-cho, Takamatsu, Kagawa 761-0396, Japan; b Fushimi Pharmaceutical Co Ltd, 307 Minatomachi, Marugame, Kagawa 763-8605, Japan; Howard University, USA

**Keywords:** crystal structure, hydrogen bonding, rare sugar, alkyl sorboside

## Abstract

Ethyl l-sorboside, C_8_H_16_O_6_, was prepared from the rare sugar l-sorbose, C_6_H_12_O_6_, and crystallized. It was confirmed that ethyl l-sorboside formed α-pyran­ose with a ^2^ *C*_5_ conformation. In the crystal, mol­ecules are linked by O—H⋯O hydrogen bonds, forming a three-dimensional network. The unit-cell volume of the title ethyl α-l-sorboside is 940.63 Å^3^ (*Z* = 4), which is about 194.69 Å^3^ (26.1%) bigger than that of l-sorbose [745.94 Å^3^ (*Z* = 4)].

## Abstract 2

The title compound was synthesized by the dehydrative condensation of α-l-sorbose and ethanol.

## Graphical abstract

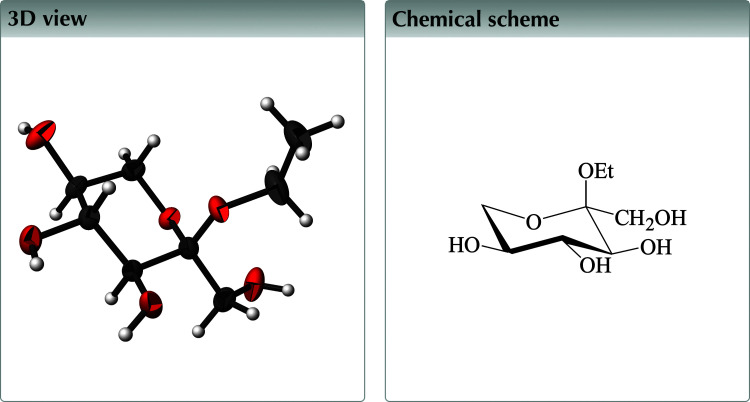

## Structure description

**Figure 1:**
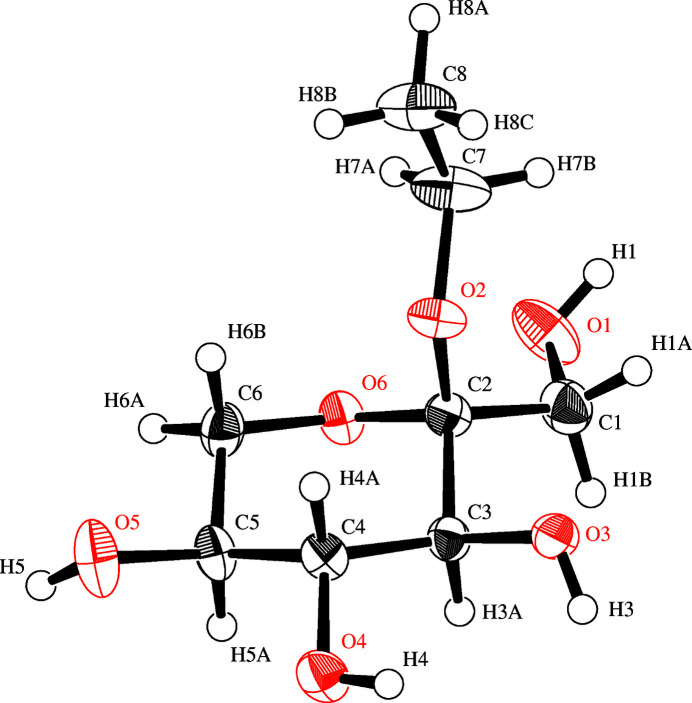
An *ORTEP* view of the title compound with the atom-labelling scheme. The displacement ellipsoids of all non-hydrogen atoms are drawn at the 50% probability level. H atoms are shown as small spheres of arbitrary radii.

The rare sugar l-sorbose is the first l-form hexose found in nature (Itoh *et al.*, 1995 ▸; Khan *et al.*, 1992 ▸; Nordenson *et al.*, 1979 ▸). Ethyl l-sorboside (Fig. 1 ▸) is an α-pyran­ose form in which the OH group located on the C-2 position in the rare sugar l-sorbose is converted into the eth­oxy group OC_2_H_5_. The mol­ecular weight of C_8_H_16_O_6_ is 208. On the other hand, the mol­ecular weight of C_6_H_12_O_6_ is 180. So, the increase in mol­ecular weight is about 16%. In contrast, the volume has increased by 26%. This point is characteristic. In other words, sorbose is highly crystalline and has a high density. On the other hand, the addition of the eth­oxy group, which is hydro­phobic, weakens inter-mol­ecular inter­actions between sugar mol­ecules, resulting in a decrease in density and an increase in volume.

**Figure 2:**
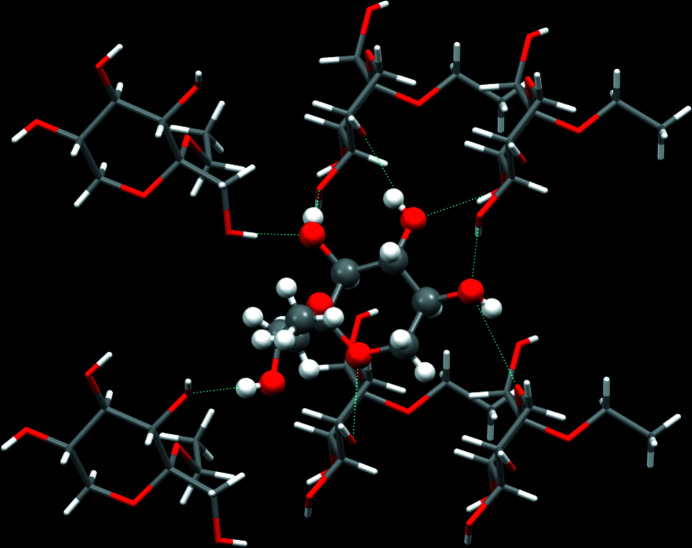
A packing diagram of the title compound, showing the hydrogen-bonding network (dotted lines).

In this study, we aimed to create a single crystal of ethyl l-sorboside. The space group is non-centrosymmetric, *P*2_1_2_1_2_1_, and there are total of four sorboside mol­ecules in the unit cell (*Z* = 4). The crystal structure of ethyl l-sorboside features a three-dimensional hydrogen-bonded network (Table 1 ▸), with each mol­ecule inter­acting with six neighbours. There are four inter­molecular hydrogen bonds and an additional intra­molecular hydrogen bond (Fig. 2 ▸).

## Synthesis and crystallization

Ethyl l-sorboside, α-sorbo­pyran­oside form, was prepared by Fischer glycosidation from l-sorbose and ethanol (Taguchi *et al.*, 2018 ▸). The Fisher method produces isomers such as α-, β-, and furan­ose. Therefore, chromatographic separation using an ion-exchange resin was performed. After the separation step, the solution was evaporated to syrup. Small single crystals were obtained by keeping the flask at room temperature. It is obvious that the synthesized ethyl α-l-sorbose is still in the l-form after dehydrative condensation, because l-sorbose is used as the starting material. The absolute structure were also confirmed by the Flack (1983 ▸) parameter.

## Refinement

Crystal data, data collection and structure refinement details are summarized in Table 2 ▸.

## Supplementary Material

Crystal structure: contains datablock(s) global, I. DOI: 10.1107/S2414314620016259/bv4033sup1.cif

Structure factors: contains datablock(s) I. DOI: 10.1107/S2414314620016259/bv4033Isup2.hkl

CCDC reference: 2046786

Additional supporting information:  crystallographic information; 3D view; checkCIF report

## Figures and Tables

**Table 1:** Hydrogen-bond geometry (Å, °)

*D*—H⋯*A*	*D*—H	H⋯*A*	*D*⋯*A*	*D*—H⋯*A*
O1—H1⋯O3^i^	0.82	2.00	2.811 (3)	169
O3—H3⋯O4^ii^	0.82	1.94	2.750 (3)	167
O4—H4⋯O3	0.82	2.52	2.879 (2)	108
O4—H4⋯O5^ii^	0.82	2.00	2.791 (2)	163
O5—H5⋯O6^iii^	0.82	2.35	2.988 (2)	136

Symmetry codes: (i) 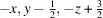 ; (ii) 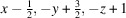 ; (iii) 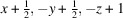 .

**Table 2:** Experimental details

Crystal data	
Chemical formula	C_8_H_16_O_6_
*M*_r_	208.21
Crystal system, space group	Orthorhombic, *P*2_1_2_1_2_1_
Temperature (K)	296
*a*, *b*, *c* (Å)	6.8203 (8), 8.6934 (10), 15.865 (2)
*V* (Å^3^)	940.63 (19)
*Z*	4
Radiation type	Cu *K*α
μ (mm^−1^)	1.09
Crystal size (mm)	0.10 × 0.10 × 0.10

Data collection	
Diffractometer	Rigaku R-AXIS RAPID
Absorption correction	Multi-scan (*ABSCOR*; Rigaku, 1995 ▸)
*T*_min_, *T*_max_	0.462, 0.897
No. of measured, independent and observed [*F* ^2^ > 2.0σ(*F* ^2^)] reflections	10373, 1721, 1602
*R*_int_	0.091
(sin θ/λ)_max_ (Å^−1^)	0.602

Refinement	
*R*[*F* ^2^ > 2σ(*F* ^2^)], *wR*(*F* ^2^), *S*	0.037, 0.090, 1.07
No. of reflections	1721
No. of parameters	127
H-atom treatment	H-atom parameters constrained
Δρ_max_, Δρ_min_ (e Å^−3^)	0.20, −0.27
Absolute structure	Flack *x* determined using 581 quotients [(*I* ^+^)−(*I* ^−^)]/[(*I* ^+^)+(*I* ^−^)] (Parsons *et al.*, 2013 ▸)
Absolute structure parameter	0.06 (12)

Computer programs: *RAPID-AUTO* (Rigaku, 2009 ▸), *SIR2014* (Burla *et al.*, 2015 ▸), *SHELXL2018/3* (Sheldrick, 2015 ▸) and *CrystalStructure* (Rigaku, 2019 ▸).

## full crystallographic data

### Crystal data

**Table:**

C_8_H_16_O_6_	*D*_x_ = 1.470 Mg m^−^^3^
*M**_r_* = 208.21	Cu *K*α radiation, λ = 1.54187 Å
Orthorhombic, *P*2_1_2_1_2_1_	Cell parameters from 9046 reflections
*a* = 6.8203 (8) Å	θ = 5.1–68.6°
*b* = 8.6934 (10) Å	µ = 1.09 mm^−^^1^
*c* = 15.865 (2) Å	*T* = 296 K
*V* = 940.63 (19) Å^3^	Block, colorless
*Z* = 4	0.10 × 0.10 × 0.10 mm
*F*(000) = 448.00	

### Data collection

**Table:**

Rigaku R-AXIS RAPID diffractometer	1602 reflections with *F*^2^ > 2.0σ(*F*^2^)
Detector resolution: 10.000 pixels mm^-1^	*R*_int_ = 0.091
ω scans	θ_max_ = 68.3°, θ_min_ = 5.6°
Absorption correction: multi-scan (ABSCOR; Rigaku, 1995)	*h* = −7→8
*T*_min_ = 0.462, *T*_max_ = 0.897	*k* = −10→10
10373 measured reflections	*l* = −19→18
1721 independent reflections	

### Refinement

**Table:**

Refinement on *F*^2^	Hydrogen site location: inferred from neighbouring sites
*R*[*F*^2^ > 2σ(*F*^2^)] = 0.037	H-atom parameters constrained
*wR*(*F*^2^) = 0.090	*w* = 1/[σ^2^(*F*_o_^2^) + (0.0379*P*)^2^ + 0.0827*P*] where *P* = (*F*_o_^2^ + 2*F*_c_^2^)/3
*S* = 1.07	(Δ/σ)_max_ < 0.001
1721 reflections	Δρ_max_ = 0.20 e Å^−^^3^
127 parameters	Δρ_min_ = −0.27 e Å^−^^3^
0 restraints	Absolute structure: Flack *x* determined using 581 quotients [(*I*^+^)-(*I*^-^)]/[(*I*^+^)+(*I*^-^)] (Parsons *et al.*, 2013)
Primary atom site location: structure-invariant direct methods	Absolute structure parameter: 0.06 (12)
Secondary atom site location: difference Fourier map	

### Special details

**Table:**

Geometry. All esds (except the esd in the dihedral angle between two l.s. planes) are estimated using the full covariance matrix. The cell esds are taken into account individually in the estimation of esds in distances, angles and torsion angles; correlations between esds in cell parameters are only used when they are defined by crystal symmetry. An approximate (isotropic) treatment of cell esds is used for estimating esds involving l.s. planes.
Refinement. Refinement was performed using all reflections. The weighted R-factor (wR) and goodness of fit (S) are based on F^2^. R-factor (gt) are based on F. The threshold expression of F^2^ > 2.0 sigma(F^2^) is used only for calculating R-factor (gt).H atoms were positioned geometrically (C—H = 0.98, 0.97 or 0.96 Å, and O—H = 0.82 Å) and refined using as riding with *U*_iso_(H) = 1.2*U*_eq_(C or O), allowing for free rotation of the OH groups.

### Fractional atomic coordinates and isotropic or equivalent isotropic displacement parameters (Å^2^)

**Table:**

	*x*	*y*	*z*	*U*_iso_*/*U*_eq_	
O1	0.0029 (3)	0.18389 (19)	0.69407 (12)	0.0427 (6)	
H1	−0.027589	0.171200	0.743583	0.051*	
O2	0.3056 (3)	0.46513 (18)	0.70305 (10)	0.0272 (4)	
O3	0.0535 (3)	0.66379 (18)	0.63075 (10)	0.0309 (5)	
H3	−0.023262	0.711551	0.600806	0.037*	
O4	0.2892 (3)	0.71889 (18)	0.48325 (11)	0.0313 (5)	
H4	0.222838	0.785460	0.506192	0.038*	
O5	0.6053 (3)	0.51250 (18)	0.46175 (12)	0.0393 (6)	
H5	0.633778	0.456529	0.421987	0.047*	
O6	0.2702 (3)	0.28944 (17)	0.59356 (11)	0.0257 (4)	
C1	−0.0118 (4)	0.3405 (3)	0.67368 (18)	0.0331 (6)	
H1A	−0.052757	0.397130	0.723326	0.040*	
H1B	−0.111982	0.353631	0.630877	0.040*	
C2	0.1802 (4)	0.4074 (3)	0.64149 (16)	0.0238 (6)	
C3	0.1421 (4)	0.5445 (3)	0.58310 (15)	0.0225 (5)	
H3A	0.050371	0.512312	0.538941	0.027*	
C4	0.3292 (4)	0.5991 (2)	0.54174 (16)	0.0242 (6)	
H4A	0.420080	0.636598	0.584961	0.029*	
C5	0.4209 (4)	0.4673 (3)	0.49524 (16)	0.0251 (6)	
H5A	0.334371	0.435115	0.449203	0.030*	
C6	0.4498 (4)	0.3347 (3)	0.55571 (16)	0.0283 (6)	
H6A	0.505486	0.248021	0.525599	0.034*	
H6B	0.541691	0.364895	0.599347	0.034*	
C7	0.3484 (6)	0.3705 (3)	0.77397 (18)	0.0434 (8)	
H7A	0.392720	0.269868	0.755600	0.052*	
H7B	0.232444	0.357408	0.808608	0.052*	
C8	0.5050 (5)	0.4492 (4)	0.8224 (2)	0.0500 (9)	
H8A	0.538256	0.388699	0.870993	0.060*	
H8B	0.618825	0.461344	0.787445	0.060*	
H8C	0.459315	0.548465	0.840200	0.060*	

### Atomic displacement parameters (Å^2^)

**Table:**

	*U*^11^	*U*^22^	*U*^33^	*U*^12^	*U*^13^	*U*^23^
O1	0.0666 (16)	0.0315 (10)	0.0301 (10)	−0.0161 (9)	0.0115 (10)	0.0011 (8)
O2	0.0373 (11)	0.0233 (9)	0.0210 (9)	−0.0029 (8)	−0.0068 (8)	0.0021 (7)
O3	0.0390 (12)	0.0297 (9)	0.0241 (9)	0.0174 (8)	0.0028 (9)	0.0013 (7)
O4	0.0418 (12)	0.0220 (8)	0.0299 (10)	0.0040 (8)	0.0069 (9)	0.0058 (7)
O5	0.0410 (12)	0.0257 (9)	0.0512 (13)	−0.0061 (8)	0.0240 (10)	−0.0085 (9)
O6	0.0287 (10)	0.0190 (8)	0.0292 (10)	−0.0008 (7)	0.0060 (8)	−0.0011 (6)
C1	0.0334 (16)	0.0325 (14)	0.0335 (15)	−0.0032 (12)	0.0054 (13)	0.0051 (11)
C2	0.0277 (14)	0.0217 (12)	0.0221 (13)	0.0013 (10)	0.0001 (11)	−0.0003 (9)
C3	0.0250 (14)	0.0213 (11)	0.0213 (12)	0.0031 (10)	0.0019 (10)	−0.0016 (10)
C4	0.0292 (15)	0.0196 (11)	0.0239 (13)	−0.0014 (10)	0.0021 (11)	−0.0001 (9)
C5	0.0260 (14)	0.0215 (12)	0.0279 (14)	−0.0037 (10)	0.0088 (11)	−0.0035 (9)
C6	0.0268 (16)	0.0236 (12)	0.0344 (15)	0.0044 (11)	0.0057 (12)	−0.0015 (10)
C7	0.066 (2)	0.0321 (14)	0.0324 (16)	−0.0072 (14)	−0.0163 (16)	0.0080 (12)
C8	0.057 (2)	0.0532 (18)	0.0398 (18)	−0.0050 (16)	−0.0183 (17)	0.0121 (14)

### Geometric parameters (Å, º)

**Table:**

O1—C1	1.403 (3)	C2—C3	1.532 (3)
O1—H1	0.8200	C3—C4	1.512 (3)
O2—C2	1.392 (3)	C3—H3A	0.9800
O2—C7	1.424 (3)	C4—C5	1.499 (3)
O3—C3	1.418 (3)	C4—H4A	0.9800
O3—H3	0.8200	C5—C6	1.512 (3)
O4—C4	1.421 (3)	C5—H5A	0.9800
O4—H4	0.8200	C6—H6A	0.9700
O5—C5	1.420 (3)	C6—H6B	0.9700
O5—H5	0.8200	C7—C8	1.483 (4)
O6—C2	1.416 (3)	C7—H7A	0.9700
O6—C6	1.419 (3)	C7—H7B	0.9700
C1—C2	1.521 (4)	C8—H8A	0.9600
C1—H1A	0.9700	C8—H8B	0.9600
C1—H1B	0.9700	C8—H8C	0.9600
			
C1—O1—H1	109.5	O4—C4—H4A	109.5
C2—O2—C7	118.2 (2)	C5—C4—H4A	109.5
C3—O3—H3	109.5	C3—C4—H4A	109.5
C4—O4—H4	109.5	O5—C5—C4	109.99 (19)
C5—O5—H5	109.5	O5—C5—C6	109.5 (2)
C2—O6—C6	113.59 (17)	C4—C5—C6	108.94 (19)
O1—C1—C2	112.8 (2)	O5—C5—H5A	109.5
O1—C1—H1A	109.0	C4—C5—H5A	109.5
C2—C1—H1A	109.0	C6—C5—H5A	109.5
O1—C1—H1B	109.0	O6—C6—C5	111.6 (2)
C2—C1—H1B	109.0	O6—C6—H6A	109.3
H1A—C1—H1B	107.8	C5—C6—H6A	109.3
O2—C2—O6	111.8 (2)	O6—C6—H6B	109.3
O2—C2—C1	115.5 (2)	C5—C6—H6B	109.3
O6—C2—C1	106.1 (2)	H6A—C6—H6B	108.0
O2—C2—C3	104.35 (19)	O2—C7—C8	106.9 (2)
O6—C2—C3	108.20 (18)	O2—C7—H7A	110.3
C1—C2—C3	110.8 (2)	C8—C7—H7A	110.3
O3—C3—C4	111.19 (19)	O2—C7—H7B	110.3
O3—C3—C2	108.60 (18)	C8—C7—H7B	110.3
C4—C3—C2	111.3 (2)	H7A—C7—H7B	108.6
O3—C3—H3A	108.6	C7—C8—H8A	109.5
C4—C3—H3A	108.6	C7—C8—H8B	109.5
C2—C3—H3A	108.6	H8A—C8—H8B	109.5
O4—C4—C5	108.6 (2)	C7—C8—H8C	109.5
O4—C4—C3	110.6 (2)	H8A—C8—H8C	109.5
C5—C4—C3	109.02 (19)	H8B—C8—H8C	109.5
			
C7—O2—C2—O6	−71.6 (3)	C1—C2—C3—C4	−172.6 (2)
C7—O2—C2—C1	49.9 (3)	O3—C3—C4—O4	−63.0 (2)
C7—O2—C2—C3	171.7 (2)	C2—C3—C4—O4	175.78 (17)
C6—O6—C2—O2	−55.5 (2)	O3—C3—C4—C5	177.69 (19)
C6—O6—C2—C1	177.7 (2)	C2—C3—C4—C5	56.5 (3)
C6—O6—C2—C3	58.9 (3)	O4—C4—C5—O5	64.3 (3)
O1—C1—C2—O2	−88.5 (3)	C3—C4—C5—O5	−175.1 (2)
O1—C1—C2—O6	36.0 (3)	O4—C4—C5—C6	−175.7 (2)
O1—C1—C2—C3	153.2 (2)	C3—C4—C5—C6	−55.2 (3)
O2—C2—C3—O3	−60.3 (2)	C2—O6—C6—C5	−60.9 (3)
O6—C2—C3—O3	−179.47 (19)	O5—C5—C6—O6	177.60 (19)
C1—C2—C3—O3	64.7 (3)	C4—C5—C6—O6	57.3 (3)
O2—C2—C3—C4	62.5 (2)	C2—O2—C7—C8	172.0 (2)
O6—C2—C3—C4	−56.7 (3)		

### Hydrogen-bond geometry (Å, º)

**Table:**

*D*—H···*A*	*D*—H	H···*A*	*D*···*A*	*D*—H···*A*
O1—H1···O3^i^	0.82	2.00	2.811 (3)	169
O3—H3···O4^ii^	0.82	1.94	2.750 (3)	167
O4—H4···O3	0.82	2.52	2.879 (2)	108
O4—H4···O5^ii^	0.82	2.00	2.791 (2)	163
O5—H5···O6^iii^	0.82	2.35	2.988 (2)	136

Symmetry codes: (i) −*x*, *y*−1/2, −*z*+3/2; (ii) *x*−1/2, −*y*+3/2, −*z*+1; (iii) *x*+1/2, −*y*+1/2, −*z*+1.
